# Field evaluation of the intermittent preventive treatment of malaria during pregnancy (IPTp) in Benin: evolution of the coverage rate since its implementation

**DOI:** 10.1186/1756-3305-4-108

**Published:** 2011-06-16

**Authors:** Tania CDA d'Almeida, Marie-Agnès Agboton-Zoumenou, André Garcia, Achille Massougbodji, Valérie Briand, Yacoubou Imorou, Gilles Cottrell

**Affiliations:** 1 Faculté des Sciences de la Santé, Cotonou, Bénin; 2 IRD UMR 216, Institut des Sciences Biomédicales Appliquées, Cotonou, Bénin; 3 Centre d'Etudes et de Recherche sur le Paludisme Asosscié à la Grossesse et à l'Enfant (CERPAGE), Cotonou, Bénin; 4 Programme National de lute contre le Paludisme, Ministère de la Santé, Cotonou, Bénin; 5 IRD UMR 216, Paris, France; 6 Faculté de Pharmacie, Université Paris Descartes, Paris, France

## Abstract

**Background:**

Malaria is an important public health problem in Africa. Pregnant women are a vulnerable population and this disease can underlie an increased risk of low-birth weight newborns (< 2500 g); these women therefore need management during pregnancy. This was previously provided by chloroquine treatment, which, because of compliance problems and drug resistance, was replaced by intermittent preventive treatment with sulfadoxine-pyrimethamine (ITPp-SP) with two single doses taken after 16 weeks of amenorrhea, at least 4 weeks apart. This protocol was recommended by the World Health Organization (WHO) in 1998 and was initiated in Benin in 2006 after its political adoption in 2004.

A retrospective longitudinal study was conducted in eight maternity hospitals in two geographical areas in Benin (in the south and north). The study investigated 2420 women who gave birth from 2005 to 2009. The antenatal cards of those women were randomly selected over 5 years with the aim of analyzing the IPT coverage in the study's maternity hospitals.

**Results:**

The rate of IPT-SP coverage evolved from 3.7% in 2005 to 87.8% in 2009 for women who had received at least one dose and from 2.7% to 68.4% from 2005 to 2009 for those who had received complete ITP (two doses). Variability in the results was observed depending on the geographical area (north/south) and the type of area (rural/urban).

**Conclusions:**

In total, application of IPT-SP 2-doses has rapidly evolved since 2005, but the objective of 80% IPT coverage has not yet been achieved throughout the country. Moreover, problems of drug shortage recurring in the field (reported by health staff) remain to be resolved.

## Background

Malaria is an important world public health problem, particularly in Africa [1]. In Benin, in 2008, malaria was the leading cause for medical consultation (39.6% of disorders encountered in consultation), hospitalization (20.1% of the causes of hospitalization), and death (15.7% of the causes of death) [2]. High-risk populations are children under 5 years of age and pregnant women in areas where malaria transmission is high [3]. Malarial infections during pregnancy are reported to cause the death of 200,000 infants every year [4] and 30 million pregnant women are estimated to live in endemic areas [5]. In high-transmission areas, the main consequences of gestational malaria are anemia in the pregnant woman and low birth weight in the newborn, which is associated with an increased risk of morbidity and mortality in the first years of life [6].

Aiming to reduce the consequences of gestational malaria, the World Health Organization (WHO) recommends a series of interventions [6-8] including Intermittent Preventive Treatment (IPT) with Sulfadoxine-Pyrimethamine (SP) in high malaria transmission areas. IPTp with SP was initiated after the massive emergence of chloroquine resistance [9-11] and the observation of poor compliance on the part of pregnant women [9,10]. ITPp consists of administering, to all pregnant women, two curative doses of an effective antimalarial drug as a preventive measure during regular prenatal consultations [6,7]. SP is administered orally in a single dose supervised beginning the 16^th ^week of amenorrhea. Several randomized clinical trials on the intermittent treatment of gestational malaria with sulfadoxine-pyrimethamine have been conducted in Africa [12-17] and SP has been shown to be the most effective medication for pregnant women in preventing gestational malaria [7]. This policy has been adopted by many African countries and was adopted in Benin in 2004, but its implementation in the field did not truly begin until 2006. A study conducted in Benin from 2004 to 2005 [18] proved the efficacy of ITP-SP compared with chloroquine.

Since the implementation of ITPp-SP treatment in Benin, several studies have evaluated the degree of ITPp coverage (the Health Demographic Study conducted in 2006 and a study sponsored by the National Malaria Control Program ([Programme National de Lutte contre le Paludisme: PNLP] in 2007) [19]. It therefore seems important to assess the ITP situation since this last study. The main objective of the present study was therefore to estimate the changes in ITPp-SP coverage since 2005 up to 2009 in the country's two zones (one in the north and one in the south).

## Methods

### Context

**Figure 1:**
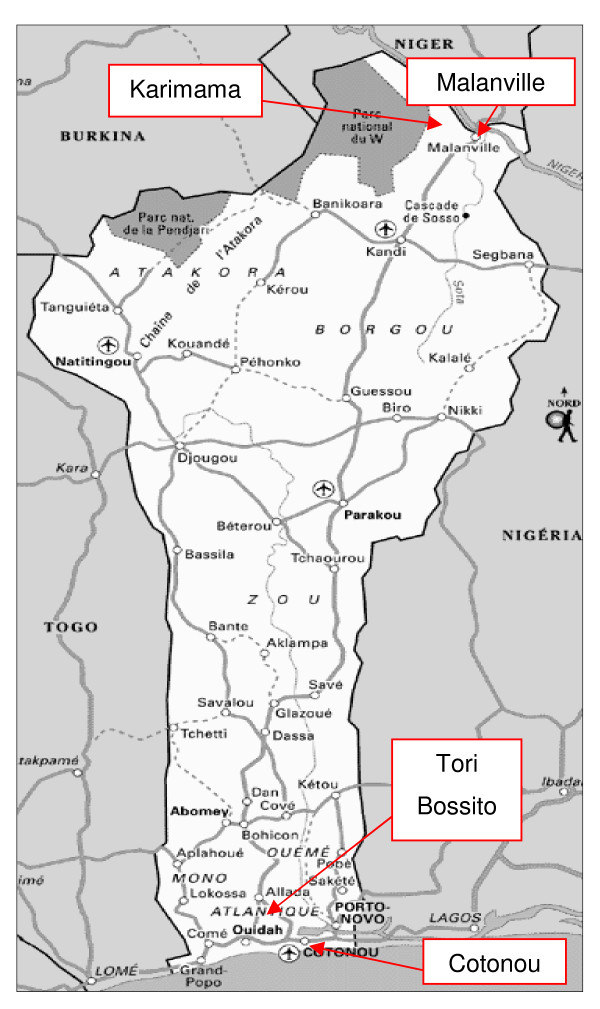
**Map of Benin**.

The study was conducted in four towns in Benin, two in the south and two in the north, with one rural town and one urban town in each area: Tori Bossito and Cotonou in the south and Malanville and Karimama in the north (Figure 1). The study was conducted in two maternity hospitals in each town for a total of four in the south and four in the north, with four located in rural areas (Tori Cada and Tori Avamè maternity hospitals in the south, Guéné and Birni Lafia maternity hospitals in the north) and four located in urban areas (the CNHU and Zogbo maternity hospitals in Cotonou in the south, and the Hôpital de Zone of Malanville and Malanville Health Center in the north). The choice of these study sites (not representative sample of all of Benin) was based on budget and ease of access considerations.

Situated in an intertropical zone, Benin has a hot and humid climate. In the south, two rainy seasons are observed (from April to July, then from October to November). In the north, the year is divided into a rainy season (May to October) and a dry season (November to April).

Transmission of malaria in Benin is perennial and malaria is rife throughout the year, with periods of recrudescence during the rainy seasons [20]. *Anopheles gambiae s. s. *has been identified as the main vector of disease transmission and *Plasmodium falciparum *as the most frequently encountered parasite (97.1%); the species *Anopheles funestus *is also present [21,22].

## Method

This was a retrospective longitudinal study consisting of collecting data from maternity registries relative to the progression of pregnancy in women who were pregnant during the 5 years between 1 January 2005 and 31 December 2009. The year 2005, before the study began, is the reference for analysis of the evolution of the IPT coverage rate.

The health staffs in hospitals were also interviewed about difficulties encountered in IPTp implementation.

### Sampling

A sample of 2420 pregnant women was constituted as follows: in each maternity hospital, a minimum of 60 maternal cards were chosen per year as the sample covering all the months of the study year, i.e., at least 300 cards for the entire duration of the study for each maternity hospital. All the year's cards were divided over the 12 months and a random selection was made for each month, i.e., five cards per month. The cards of women who did not give birth at the maternity hospital or those whose pregnancy was not monitored at the hospital were excluded from the random selection.

### Statistical analyses

The ITP coverage rate was studied and defined in two ways:

- the proportion of women who had received at least one ITP dose during their pregnancy, designated as "ITP coverage-at least one dose";

- the proportion who had received both ITP doses recommended, designated as "ITP coverage-two doses."

After a descriptive study (comparison of the progression of coverage between the maternity hospitals), the analysis consisted in logistic regression modeling of the probability of a women receiving either at least one dose of ITP or two doses of ITP in relation to certain factors (explanatory variables):

- the geographical area (north, south),

- the type of town (urban or rural),

- the study period (2005, 2006-2007, 2008-2009),

- the number of consultations (< four prenatal consultations, ≥ four prenatal consultations),

- parity (primipara, secundipara, multipara),

- gestational complications (with or without complications),

- season (dry, rainy),

- the woman's age (< 18 years, 18-34 years, ≥ 35 years).

A univariate analysis was performed between the variable to explain the above-mentioned cofactors, then the significant covariates were selected to construct the final model obtained after multivariate analysis.

All the analyses were performed using STATA Version 11.

### Ethics

IRD ethical committee and the National Research Ethical Committee of Benin gave their clearance for the study (Comité National Provisoire d'Ethique pour la Recherche en Santé, reference number IRB00006860).

## Results

### Study population characteristics

The mean age of the women was 26 years (standard deviation [SD] = 5), the minimum age was 12 years, and the oldest woman was 45 years old.

The primiparae accounted for 19.4% of the women in the study. The average parity was four pregnancies (SD = 2), varying from 1 to 13.

The mean number of prenatal consultations was four (SD = 2), varying from 1 to 13 consultations, with 72.1% of the women having attended at least four consultations.

The first prenatal consultation occurred at a mean 16 weeks (SD = 8) of amenorrhea (median = 14 weeks). The first consultation took place during the first trimester of pregnancy (i.e., before the 16^th ^week of amenorrhea) for 56.0% of the women.

### Overall ITP-SP coverage in pregnant women from 2005 to 2009

**Figure 2:**
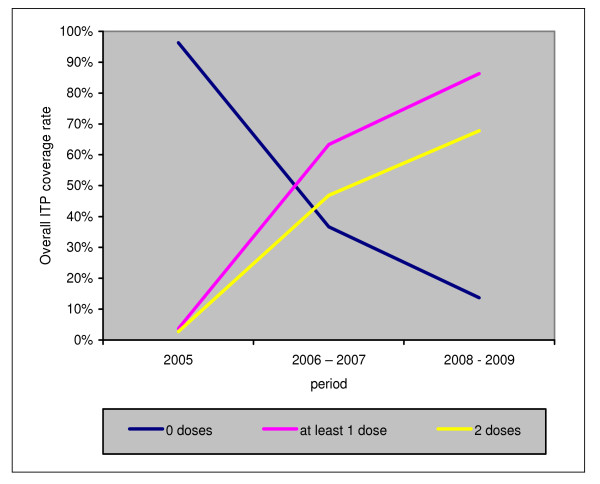
**Overall progression of ITP intake in pregnant women from 2005 to 2009 in the study's maternity hospital**.

Figure 2 represents the changes in ITP-SP from 2005 up to 2009, in all the study's maternity hospitals. It shows that ITP started very slowly in 2005. The ITP coverage rate showed a rapid increase after 2006, then a less rapid increase after 2008. From 2006 to 2009, a constant gap between the progression of the coverage rate of "ITP-at least one dose" and the rate of "ITP-two doses" can be observed. In 2009, again 15% of the pregnant women registered in consultation did not receive a single dose of ITP and 30% did not receive the two doses required.

### ITP coverage rate per maternity hospital from 2005 to 2009

**Figure 3:**
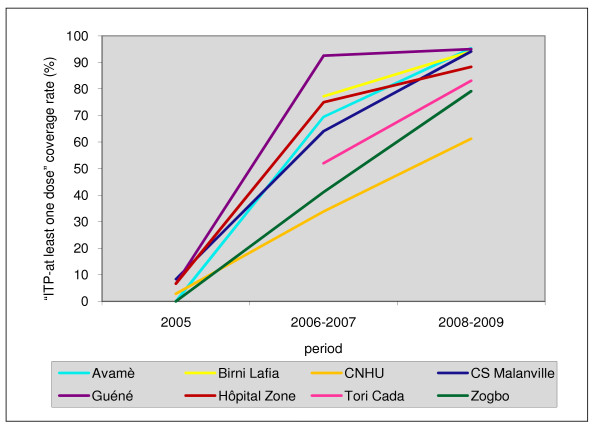
**Progression of "ITP-at least one dose" coverage rate in pregnant women in each maternity hospital from 2005 to 2009**.

In 2005, as indicated in Figure 3, the level of "ITP-at least one dose" (ITPp 1) use was low in all the maternity hospitals (< 10%). During the 2006-2007 period, the rate of "ITP-at least one dose" coverage varied between 33.9% (CNHU) and 92.6% (Guéné). During the 2008-2009 period, the rate of "ITP-at least one dose" coverage improved and varied between 61.3% (CNHU) and 95.3% (Avamè). In all cases, good progression was observed over time, bringing to light two groups. Some maternity hospitals experienced a rapid start, above 60% "ITP-at least one dose" coverage during 2006-2007 and then evolved less quickly: the maternity hospitals in the north and Avamè. The others, located in the south, evolved more slowly, in a linear fashion and in relation to time: the CNHU, Zogbo, and Tori Cada maternity hospitals.

**Figure 4:**
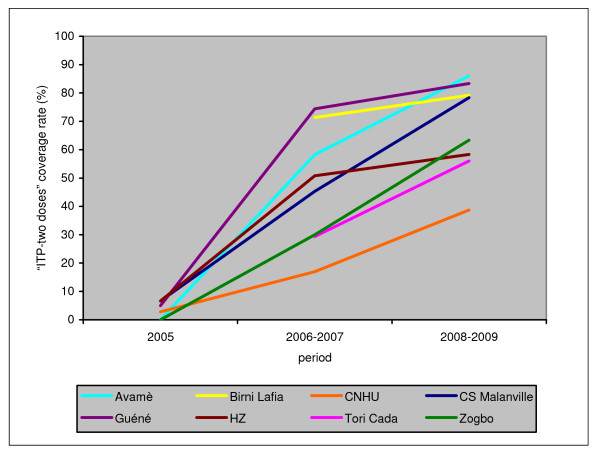
**Progression of "ITP-two doses" coverage rate in pregnant women in each maternity hospital from 2005 to 2009**.

Figure 4 shows the progression of the "ITP-two doses" coverage rate in the study's eight maternity hospitals. In 2005, the" ITP-two doses" coverage rate was very low in the maternity hospitals (≤ 10%) and nil in Avamè and Zogbo. During 2006-2007, more than half of the women received two doses in four maternity hospitals (Guéné, Avamè, Birni Lafia, and the hospital in the Malanville area), then the rate evolved more quickly during the last period (2008-2009).

In the four other maternity hospitals, progression was slower between 2005 and 2006-2007, but it remained constant over time to such an extent that by the end of the study the "ITP-two doses" coverage rate at the Malanville Health Center, for example, caught up with the most advanced maternity hospitals at the beginning of the study. The CNHU here again showed a specific profile, with relatively slow but constant growth. In 2008-2009, the "ITP-two doses" coverage rate showed high variability between the different maternity hospitals, ranging from 39% for the CNHU to 86% for Avamè.

**Figure 5:**
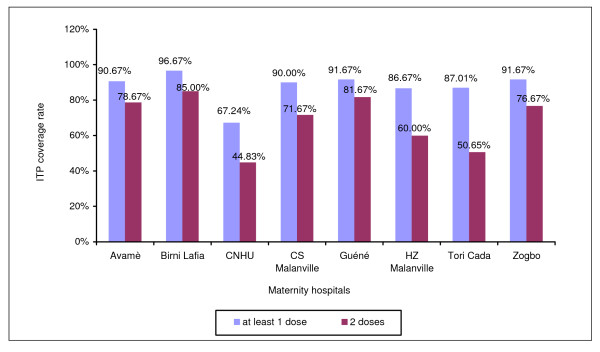
**ITP coverage rate in all the maternity hospitals in 2009**.

Figure 5 shows the ITP-SP coverage rate in all the maternity hospitals for the year 2009, the last year of the study. The proportion of women who had received at least one dose varied from 67% (CNHU) to 97% (Birni Lafia), with a percentage above 80% for seven maternity hospitals out of eight. However, the "ITP-two doses" coverage rate was lower in all the maternity hospitals, fluctuating between 45% (CNHU) and 85% (Birni Lafia). Guéné and Birni Lafia were the two maternity hospitals having more than 80% coverage for "ITP-two doses." In 2009, 87.8% of the women received at least one dose of SP and 68.4% of them received the two required doses. The CNHU was the center with the lowest coverage rates.

### Factors influencing administration of ITP-SP in the maternity hospitals from 2005 to 2009

Table 1 shows the results of the final multivariate logistic regression model of the variable "ITP administration" on only the significant covariates. This analysis demonstrates that for the entire study, beyond the period whose very high odds-ratio reflected the rapid progression since 2005, certain factors were significantly associated with ITP intake:

**Table 1:** Significant factors in ITP-SP administration for the entire study

Variables		Numbers of patients	Odds ratio	Confidence interval	*P*-value
Geographical area	Urban	1177			<10^-4^
	Rural	1243	2.77	[2.20;3.48]	
Geographical location	North	1119			<10^-4^
	South	1301	0.26	[0.20;0.33]	
Study period	2005	406			<10^-4^
	2006-2007	974	59.46	[34.22;103.30]	
	2008-2009	1040	257.73	[145.22;457.41]	
ANC	< 4	675			<10^-4^
	≥ 4	1745	1.85	[1.46;2.33]	

NB: ANC = Antenatal care

- the geographical area (on average a better chance of receiving ITP for pregnant women living in the north);

- type of town (on average a better chance of receiving ITP for pregnant women living in a rural area compared to those living in urban areas),

- the number or prenatal consultations (on average a better chance of receiving ITP for pregnant women who had undergone at least four prenatal consultations).

### Difficulties encountered by the staff

The main difficulty for the maternities staff is the shortage of medicine in maternity clinics. By this way, some women did not receive ITPp at a correct time.

## Discussion

The malaria prevention strategy for pregnant women with sulfadoxine-pyrimethamine treatment recommended by the WHO in 1998 was adopted by the Beninese government in 2004. However, it was only implemented in the field starting in 2006. The present study covered the period from 2005 to 2009, including all the available data since the year preceding the protocol's implementation. The objective was to assess the ITP-SP coverage rate in two regions in the south and north of the country from its implementation until 2009. The results showed that the ITP coverage rate had evolved rapidly since implementation of the protocol even if this varied in the study's eight maternity hospitals. Since these were factors associated with ITP administration, the results show that ITP administration may be associated with the type of town (rural or urban) and the geographical area (north or south), as well as the number of prenatal consultations.

Overall, in 2005 we observed a low "ITP-at least one dose" and "ITP-two doses" coverage rate, confirming that in this period the administrative decision had not been applied in the field. During this period, 92.6% of the women in the study still received chloroquine treatment. The rapid progression of the coverage rate observed after start-up in 2006 is probably the consequence of awareness campaigns and training maternity hospital personnel in the application of this new protocol. This progression continued for the 2008-2009 period, although at a slower pace.

However, this overall progression hides substantial disparity between the maternity hospitals. We noted a globally faster progression in maternity hospitals located in the north of Benin. A possible explanation would be that ITP-SP may have been adopted earlier in this area and therefore that personnel training may have taken place earlier than in the south.

In total, in 2009, 3 years after starting up ITP-SP, all the maternity hospitals in the north (Birni Lafia, Guéné, Hôpital de zone, and Health Center) showed more than 80% coverage in "ITP-at least one dose." Also in the south, the Tori Cada, Avamè, and Zogbo health centers also showed a coverage rate higher than 80%. Only the CNHU was below this percentage, with a 67% "ITP-at least one dose" coverage rate. The maternity hospitals in the south, except for CNHU, were no longer behind those in the north, as observed in 2006-2007.

However, in 2009, only two maternity hospitals out of eight reached the 80% "ITP-two doses" coverage threshold, as established as the objectives of the WHO Roll Back Malaria [23] program and Benin's PNLP [24] for 2010. The coverage rate varied from 45% at the CNHU to 85% at Birni Lafia in 2009. Overall, the coverage rate was 68% in 2009.

The difference between the number of women receiving at least one dose and those having received two does of SP could be explained by several factors:

- too few consultations or consultations too far apart, precluding SP administration (which cannot be administered after the 36^th ^week of amenorrhea);

- a stock shortage of the drug in certain maternity hospitals;

- late consultations for certain pregnant women (the first prenatal consultation occurred after 32 weeks for 5% of women), thus leaving insufficient time for the second administration before giving birth;

- abortions or premature births occurring before the second administration;

This study also showed that the CNHU was the center where coverage ("ITP-two doses" and "ITP-at least one dose") was the lowest. Various factors can explain this:

- First, the CNHU receives a large number of pregnant women and may have been hit more severely by the stock shortage of SP than expected. In this case, it was observed that women sometimes received another antimalarial such as chloroquine, proguanil or the association of the two antimalarials, for example. This may protect the pregnant woman but does not match the national or WHO recommendations;

- A greater proportion of women consulting at the CNHU have an at-risk pregnancy than in the other health centers. This is the case, for example of sulfamide allergies and HIV seropositivity for which a different protocol is recommended [20].

Comparable studies have been conducted in Benin and in other African countries.

- In Benin, the PNLP evaluated the strategies of malaria control during pregnancy was conducted in 2007, in a transversal study throughout the country. It showed that 60.7% of the women surveyed in healthcare training and 66.6% of the women in the general population had received ITP-SP appropriately (two doses), for a total of 63.7% [19]. This percentage is close to that found in the present study for the overall population of pregnant women: 59.0% in 2007. This study also reported that the availability of SP is unstable in the university-affiliated hospitals and the departmental hospitals [19]. However, it should be noted that the ITP coverage rate was calculated based on data collected on the maternal cards in our study, whereas it was based on interviews with women in the PNLP study.

- Several studies, in the Gambia in 2008 [25], two others in Kenya in 2004 [26] and 2008 [27], and in Malawi in 2000 [28], showed less rapid progression of the ITP-two dose coverage rate than in the present study.

- The authors suggest that these results may be related to poor awareness on the part of women, most of whom seemed not to return for the follow-up doses, or problems of geographic accessibility of the center, or socioeconomic problems [27].

The differences between these studies and ours could also be related to the methods used. First of all, the study periods were different, and for the studies conducted in Kenya and Malawi, the data were obtained from interviews of pregnant women, certain of whom could have forgotten that they had received ITP-SP, resulting in an underestimation of ITP coverage. In addition, in Benin, where the coverage rate seems better than that found in other studies, SP is subsidized and distributed free.

Another factor is that in the present study the coverage rate reflects women who came for prenatal consultations and does not take into account women who were not seen in these visits. In 2008 for example, the coverage rate in prenatal consultations was estimated at 76.9% for the Alibori department in the northern area and 89.2% for the Atlantique department where the Tori Cada and Tori Avamè maternity hospitals are located. Our protocol therefore overestimates the real ITP coverage rate to a certain extent.

Except for the study period, the geographical area (with a higher probability of receiving ITP in the north than in the south), and the type of town (with a higher probability of receiving ITP in rural areas than in towns), the results of this study show that women who had had at least four prenatal consultations had a better chance of receiving ITP than the others. This result confirms the WHO recommendations [7] and the PNLP's recommendations as well [20], according to which pregnant women should have at least four prenatal consultations, three of which after active fetal movements (or after the 16^th ^week of amenorrhea). If SP was administered during these last three prenatal visits, a large proportion of pregnant women would receive the two recommended doses.

## Conclusions

All in all, the administration of ITP-SP for malaria has clearly advanced in the maternity hospitals investigated in this study, even if the study was based only on women who attended prenatal consultations during their pregnancy. A certain variability exists between the north and the south, between rural and urban areas, and even between the different maternity hospitals in the same town. The availability of this drug is certainly acceptable in some centers. If the number of women received in prenatal consultations remains far from the expected number of women, the coverage rates should be even less than what was found. This study in the two regions where maternity hospitals were selected cannot be representative of all of Benin, but it provides a first evaluation of how the ITP-SP situation is evolving. This observation implies significant actions to intensify the administration of SP as the preventive treatment for malaria in pregnant women.

## Competing interests

The authors declare that they have no competing interests.

## Authors' contributions

All the authors read and approved the final version of the manuscript.
